# Impact of the Alien Aquatic Plant *Ludwigia hexapetala* on the Native *Utricularia australis*: Evidence from an Indoor Experiment

**DOI:** 10.3390/plants12040811

**Published:** 2023-02-10

**Authors:** Emanuele Pelella, Beatrice Questino, Simona Ceschin

**Affiliations:** 1 Department of Sciences, University of Roma Tre, Viale G. Marconi 446, 00146 Rome, Italy; 2 NBFC—National Biodiversity Future Center, 90133 Palermo, Italy

**Keywords:** water primrose, aquatic bladderwort, invasive macrophyte, non-native versus native plants, interspecific competition, allelopathy, freshwater ecosystem

## Abstract

*Ludwigia hexapetala* is an alien aquatic plant considered highly invasive in Europe since it alters freshwater habitats by forming dense mats both in water and along banks, outcompeting natives. *Ludwigia* effects on the native carnivorous plant *Utricularia australis* were investigated here. A 21-day indoor experiment was performed by setting up some separate tests in which *Utricularia* was made to grow both alone (control tests) and together with *Ludwigia* (arena tests). Water chemical and physical parameters and growth and morphological traits of *Utricularia* and *Ludwigia* were analysed weekly. Water samples were also analysed by UV-visible spectra to verify allelochemical (quercitrin) production by *Ludwigia*. In arena tests, oxygen concentration and pH were lower and conductivity higher than in control tests. *Utricularia* grew significantly less in arena tests in both shoot length and internode number, and its fresh weight, trap number and internode length decreased more than the control. Quercitrin was found only in arena tests as an allelochemical product released by *Ludwigia*. Overall, this study demonstrated *Ludwigia* significantly alters water parameters and negatively affects the growth of *Utricularia*, showing aggressive and competitive behaviour against this native species. Such evidence suggest that the widespread of *L. hexapetala* can represent a serious threat to the conservation of native plant diversity occurring in the freshwater habitats it invades.

## 1. Introduction

Biological invasions are one of the most important drivers of native biodiversity loss and ecosystem degradation worldwide [1]. Invasive alien plants, which are highly adaptable species with high reproductive capacity, often outcompete native species, causing deterioration of native biodiversity and alteration of the structure and ecological balances in the plant communities of the invaded ecosystem [2,3,4]. Consequently, there is a progressive impoverishment of those ecosystems [5], whose conservation status becomes more and more compromised [6]. Although there is a general awareness of the negative effects of invasive alien plant species on native communities and ecosystems, studies concerning the impacts of such species in Europe and, more specifically in Italy, are still quite scarce [7]. Therefore, filling this knowledge gap should be one of the priorities in scientific research and conservation actions. 

Freshwater ecosystems are particularly susceptible to biological invasions because of their intrinsic vulnerability and peculiarity [8,9]; indeed, Lazzaro et al. found that freshwater habitats in Europe are among those invaded and impacted by the highest number of alien plant species among all Natura 2000 habitats [7]. One of the alien aquatic plant that can represent a serious threat to the conservation of such habitats in Europe is the American *Ludwigia hexapetala* (Hook. and Arn.) Zardini, H.Y. Gu and P.H. Raven. Other related alien species of the genus *Ludwigia*, such as *L. peploides* (Kunth) P.H. Raven and *L. grandiflora* (Michx.) Greuter and Burdet have been included since 2016 in the List of Invasive Alien Species of Union concern [10], which implies that these species cannot be imported, cultivated, commercialised, or intentionally released into the environment in the whole of the European Union. The fact that *L. hexapetala* seems to show very similar morphology, ecology and reproductive capabilities to *L. peploides* [11,12] and that some authors point to *L. hexapetala* as a subspecies of *L. grandiflora* [13] suggests the possibility that this species may also soon be included in the Union list. 

*Ludwigia hexapetala* was first observed in the wild in France towards 1830 [14], and its increased use as an ornamental plant during the 20th century has accelerated its expansion in other European countries [15], among which Italy. Here, it was first reported in 1934 in the north, under the synonym *Jussiaea repens* L. [16], from which it expanded to other northern-central regions [17,18]. *Ludwigia hexapetala* shows two distinct growth forms characterised by heterophylly: a horizontal growth form with buoyant, vegetative leaf rosettes and a vertical form with emergent flowering stems [19]. Once established, it can invade both aquatic and bank zones of lakes, ponds, rivers, and ditches, producing dense vegetative mats that affect drainage and water quality, reducing biodiversity and establishing anoxic conditions that are unfavourable for aquatic life [20,21,22]. In addition, some studies showed that *L. hexapetala* has an intense allelopathic activity that enables it to produce and release in the surrounding environment some chemical substances (allelochemicals) that hinder the seed germination and limit the growth of the other aquatic plants, especially natives [15,19]. Indeed, the allelopathy strategy can favour the establishment and spread of alien plants to the detriment of the native species [15,23,24]. The allelochemicals can have a direct or indirect effect on other plant species and can be released in different modes, such as volatilisation, plant decomposition, or root exudation, as in the case of *Ludwigia* [25]. 

In the freshwater habitats that *L. hexapetala* could colonise, the survival of many native aquatic plants, already facing local extinction, could be seriously threatened by the arrival of this species. One of these native species is the bladderwort *Utricularia australis* R. Br., classified as a near-threatened species (NT) in Italy based on IUCN categories [26,27]. *Utricularia australis* is a carnivorous aquatic plant that produces free-floating populations in sunny, oligo-(meso)trophic and shallow freshwater habitats [28]. It is characterised by capilliform leaves interspersed with modified leaves for carnivory (traps) that act by trapping small aquatic organisms [28]. Its distribution is gradually contracting in Italy, and it has been observed that in some locations in central Italy, where *L. hexapetala* is becoming more and more dominant, it is completely disappearing [29].

Within this background, the present study focused on assessing the impact of the alien *L. hexapetala* on the native *U. australis*, assuming that this occurs both directly, by affecting its growth and development of some of its vegetative traits, and indirectly, by deteriorating the water quality of the environment in which *U. australis* grows. An indoor experiment was performed by setting up some separate tests in which *Utricularia* was made to grow both alone and together with *Ludwigia*; some water parameters and growth and morphological-structural traits of the two aquatic plants were monitored and measured over time.

## 2. Results and Discussion

### 2.1. Water Chemical and Physical Variations

**Figure 1:**
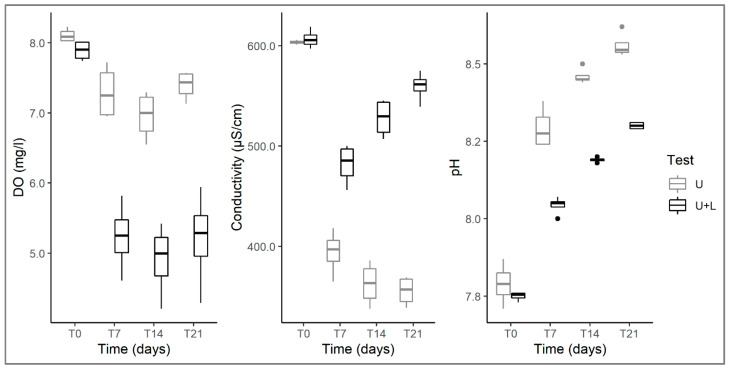
Changes in dissolved oxygen (**left**), conductivity (**centre**) and pH (**right**) at different times (T0, T7, T14, T21) in control tests with *U. australis* alone (U) and in plant arenas (U+L) in which *U. australis* grow with *L. hexapetala*. The box plots show the median (line across the box), the upper and lower quartiles (the upper and lower parts of the box), values outside the quartiles (the whiskers) and the outliers (circle points that extend beyond the whiskers).

A significant reduction in dissolved oxygen over time was observed both in control and plant arena tests (Table 1), but it was significantly more evident in the arena test, where *U. australis* and *L. hexapetala* grew together (Figure 1). In fact, at the end of the experiment (at T21), dissolved oxygen in the arena test was 42% lower than in the control. This result suggests that *L. hexapetala* is very efficient in absorbing dissolved oxygen from water, severely limiting its availability to the other aquatic plants co-occurring with it, in this case, *Utricularia*. Such efficiency is to be related to its ability to produce whitish, spongy-looking root appendages (pneumatophores) that increase the root surface area that can absorb oxygen, as has also been pointed out in other studies [13,30]. Indeed, *Ludwigia* samples equipped with these pneumatophores were observed during this experiment.

The pH levels increased steadily in both the control and plant arena tests throughout the experiment, but these increases were significantly more pronounced where *Utricularia* grew without *L. hexapetala* (Table 1; Figure 1). It is known that pH levels in water are altered by the growth of aquatic plants. Generally, through the photosynthesis of submerged plants, CO_2_ is subtracted from the water, reducing the concentration of carbonic acid (H_2_CO_3_), which dissociates into a hydrogen ion (H^+^) and bicarbonate (HCO_3_^−^), leading to a reduction in the concentration of H^+^ ions and, thus, an increase in pH values [31,32,33]. During the experiment, it was observed that *L. hexapetala* changed its growth form ranging from floating aquatic rosettes to emergent, elongated stems with leaves completely out of the water. Therefore, while submerged *Utricularia* shoots sourced CO_2_ needed for photosynthesis from the water, in the case of *Ludwigia*, the amount of CO_2_ required would mainly be sourced from the air rather than water. Consequently, any pH variation in the water is mainly to be attributed to the photosynthetic activity of *U. australis*; therefore, the reduced pH increase in the tests with *L. hexapetala* could be the result of limited photosynthetic activity of *U. australis*, which is affected by the presence of *L. hexapetala*.

Water conductivity, a factor dependent on ionic components dissolved in water, differed significantly between control and arena tests during the experiment (Table 1). Although a reduction in conductivity was observed in both tests at T7, conductivity began to increase significantly after the first week in the arena tests while continuing to show a steady decline in the controls. By the end of the experiment (T21), water conductivity was 57% higher in the arena tests than in the control (Figure 1). The reduction in conductivity in the control can be explained considering that aquatic plants, in this case, *Utricularia*, usually absorb minerals from the water during their growth [34]; in addition, their photosynthesis increases water pH levels, creating conditions favourable to the precipitation of calcium carbonate [33,35], therefore reducing water conductivity. Differently, the conductivity increase that has been observed after the first week in arena tests can be explained by taking into consideration two different aspects. Firstly, in the arena, unlike the control tests, *U. australis* showed a significant biomass loss (as described below) and tissue decomposition. Generally, the degradation of plant tissues involves the release of cellular contents into water, including mineral salts and ions, thus causing an increase also in conductivity [36]. The conductivity increase in arena tests could also be a consequence of the release in the water of allelopathic substances that could indirectly alter some chemical water parameters, including conductivity. In fact, *L. hexapetala* releases in the water a mixture of allelochemicals, such as saponins, tannins, polyphenols, alkaloids, linolenic acids, flavonoids, and glycosides, such as quercitrin, prunin and myricitrin [19,37]. Flavonoids, tannins, and phenolic acids can alter cell membrane permeability in plant species, negatively affecting their nutrient uptake and water conductivity [38,39,40,41,42]. This could mean that individuals of *U. australis* growing together with *L. hexapetala* were affected by the presence in water of such allelopathic substances, which could have hindered the native species in its capability to absorb minerals and ions from the water, resulting in absence of any decrease in water conductivity.

**Figure 2:**
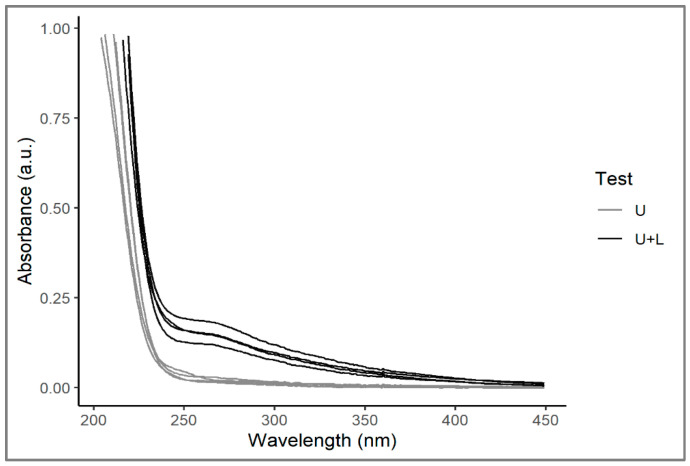
UV-visible spectra of water samples taken from control (U) and plant arena (U+L) tests. For the explanation of test acronyms, see legend of Figure 1.

UV-visible spectrophotometric profiles of water samples taken from the two different test groups showed significant differences. Samples collected in arena tests revealed a small peak in absorbance around 260 nm, which was instead absent in control tests (Figure 2). This peak is comparable to the UV-visible spectra observed for quercitrin [43,44], a glycoside that shows two peaks in absorbance at 256 and 350 nm. As the quercitrin detected by these analyses was in a very diluted form, it is obvious that the absorbance peaks observed here could not have been as obvious as those that would be obtained by analysing pure quercitrin. Since quercitrin is one of the main components of the allelopathic mixture produced by *L. hexapetala* [19], this suggests that the alien species exhibited allelopathic activity during the experiment.

### 2.2. Plant Parameter Analyses

**Figure 3:**
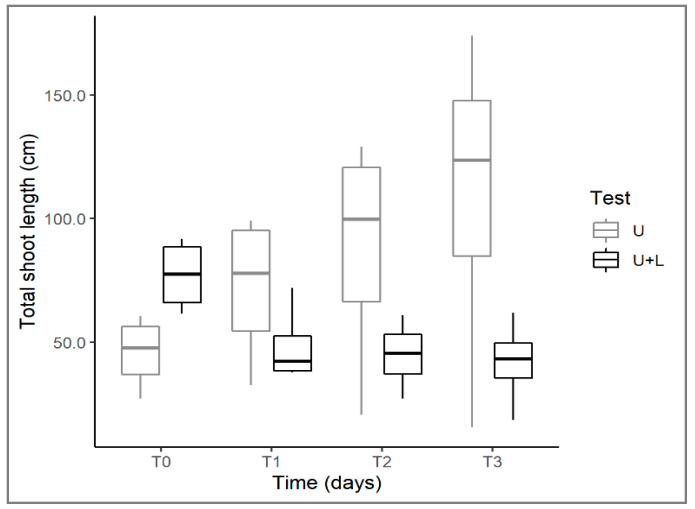
Changes in total shoot length of *U. australis* in control (U) and plant arena (U+L) tests at different times (T0, T7, T14, T21). For the explanation of boxplots and test acronyms, see legend of Figure 1.

*Utricularia australis* showed an increase in total shoot length in control tests over time; conversely, there was a decrease in arena tests (Table 1), meaning that in the presence of the alien species, its growth was limited (Figure 3). Moreover, where *L. hexapetala* was present, *U. australis* shoots tended to fragment into several pieces, with degeneration of shoot sections, which could be an effect of the presence of the invasive *Ludwigia* and its allelopathic action against *Utricularia*.

**Figure 4:**
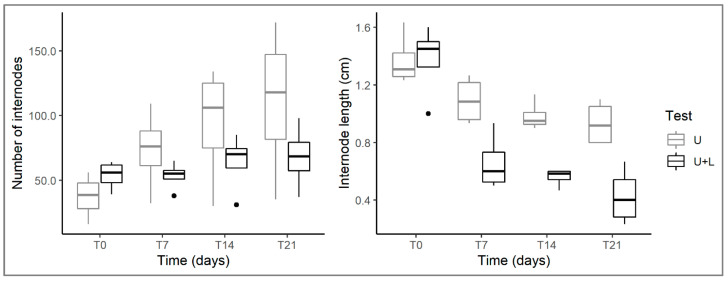
Changes in internode number (**left**) and internode length (**right**) of *U. australis* in control (U) and plant arena (U+L) tests at different times (T0, T7, T14, T21). For the explanation of boxplots and test acronyms, see legend of Figure 1.

The number of internodes of *U. australis* increased during the experiment both in control and arena tests. However, this increase was much lower in the arena test, where the mean number of internodes was 38% lower than in the control at the end of the experiment (Table 1; Figure 4), indicating slower growth when the native plant is co-occurring with the alien one. Marginal R^2^ (0.26) was much lower than conditional R^2^ (0.86), indicating that much of the variance was explained by differences at the microhabitat level between the tanks where the experiment was carried out. This slower growth of *U. australis* in the test with *L. hexapetala* could be another indicator that the native plant is negatively affected by the competition with the alien. These observations are further confirmed by data on internode length in *U. australis*. In fact, although generally there was a decrease in internode length throughout the experiment, both the arena test and the interaction term, representing the effect of the presence of *Ludwigia* over time, had a significant influence (Table 1). This means that in arena tests, *Ludwigia* exerted a negative impact over time on the growth of *U. australis*, which showed rapidly shrinking internodes, a variation that was much less noticeable in the control tests. The overall reduction in internode length in *U. australis* over time, both in control and arena tests, could be caused by a stress condition in the native plant due to the mechanical handling to which the samples were subjected at each experimental time point to measure the different biological parameters.

**Figure 5:**
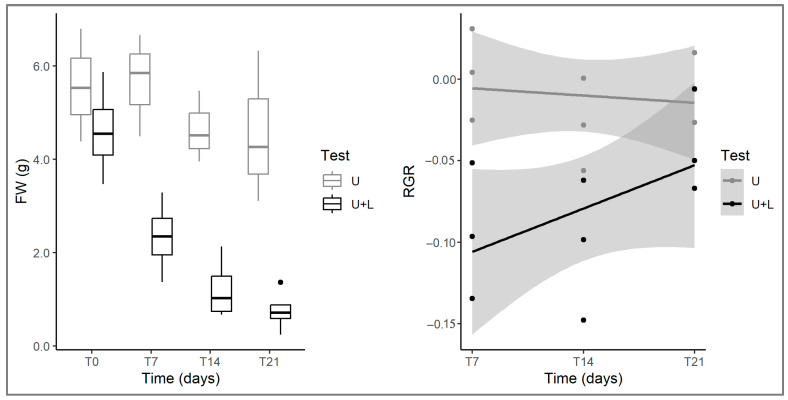
Changes in *U. australis* fresh weight (boxplots on the left) and RGR (regression lines on the right) in control (U) and plant arena (U+L) tests at different times (T0, T7, T14, T21). For the explanation of boxplots and test acronyms, see legend of Figure 1.

Data on fresh weight (FW) showed an overall decrease in *Utricularia* biomass during the experiment both in control and arena tests, which, as stated previously, could be a consequence of mechanical stress to which *Utricularia* shoots were subjected weekly. Nevertheless, *Utricularia* samples grown together with *L. hexapetala* showed much lower values of FW, and a faster decrease in weight, which means that the presence of the alien species constituted an additional stressor that slowed down the growth of the native species (Table 1; Figure 5). These observations are further confirmed by data on relative growth rate (RGR). In fact, although *Utricularia* RGR did not vary significantly over time (*p* > 0.05), there was a significant difference between control and arena tests (Table 1). In control tests, *Utricularia* RGR values were slightly negative, while in arena tests, they were much more negative and stayed negative throughout the experiment. This means that the native species suffered a much greater reduction in vegetative growth and new biomass production when mated with the alien *Ludwigia*. 

**Figure 6:**
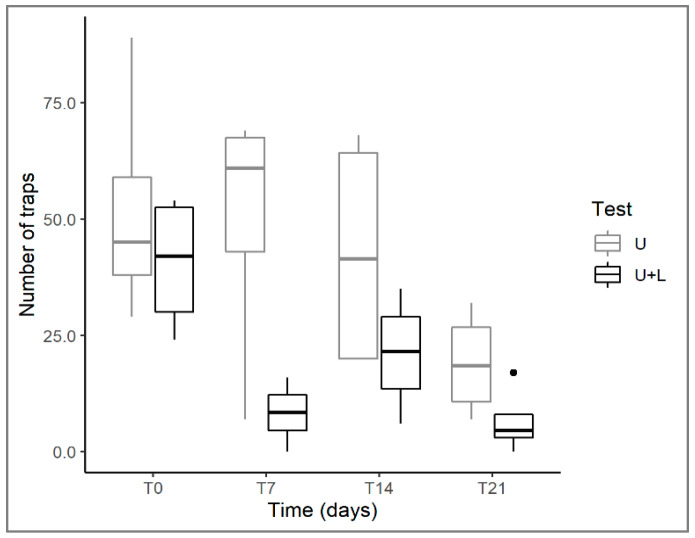
Changes in *U. australis* in number of traps in control (U) and plant arena (U+L) tests at different times (T0, T7, T14, T21). For the explanation of boxplots and test acronyms, see legend of Figure 1.

The number of traps decreased significantly during the experiment in both control and arena tests, although these data presented a high variance that was not fully explained by the model (Table 1). However, there was a significant difference between the two tests since where *U. australis* grew with *L. hexapetala*, the decrease in the number of traps was much more evident; in particular, at T21, *Utricularia* produced 66% fewer traps in the arena than in the control tests (Figure 6). The loss of traps in all tests over time could be related to a condition of stress in *U. australis*, as discussed above. However, the significantly greater loss of traps in the presence of *Ludwigia* again highlights how this alien species has a negative effect on several structural traits of the native plant. 

Overall, it is clear that the alien *L. hexapetala* has a negative impact on the growth and morphological and structural traits of the native *U. australis*. These results generally agree with those obtained in the study of Thiébaut et al. [19], in which the growth of another native species, *Ceratophillum demersum* L., was severely restricted by the presence of allelopathic substances released by *L. hexapetala*. In contrast, in another similar study [45], *L. hexapetala* did not appear to affect the growth of the native species, *Mentha aquatica* L., although some negative effects were recorded, such as a decrease in root biomass caused by the allelopathic activity of *Ludwigia*. The fact that the allelopathic substances produced by *Ludwigia* cause more pronounced and varied negative effects on *U. australis* and *C. demersum* than *M. aquatica* might suggest that some aquatic native plants are more sensitive than others to the presence of this alien plant. However, apart from this different susceptibility of native species, several authors recognise this allelopathic activity as a competition strategy that *L. exapetala* adopts against native species, often enabling it to outcompete them, as occurred in this study, and thus also colonise new areas outside its range [15].

**Figure 7:**
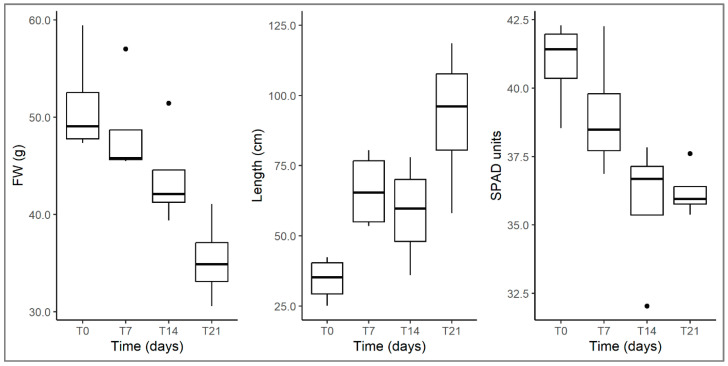
Changes in fresh weight (**left**), total shoot length (**middle**) and SPAD (**right**) in *L. hexapetala* in arena tests at different times (T0, T7, T14, T21). For the explanation of boxplots, see legend of Figure 1.

In parallel with the investigations on *U. australis*, analyses of the growth and morphological traits of the alien *L. hexapetala* were carried out to evaluate its health and growth status under the experimental conditions established. The results indicated that its biomass decreased during the experiment, but its total length increased (Table 1; Figure 7). This reflects the growth form changes occurred in *L. hexapetala* throughout the experiment, shifting from aquatic, buoyant rosettes to elongated leafy stems emerging from the water. Therefore, the plant did not produce new biomass but distributed it differently, mostly investing in height growth, confirming what is also reported in the literature [46]. This finding is also confirmed by data on *Ludwigia* RGR, which, while not varying significantly during the experiment, showed negative values indicating a trend towards biomass loss. Also, SPAD values decreased, suggesting a condition of stress in the tested individuals of *L. hexapetala* (Table 1; Figure 7). In fact, the SPAD index has been widely used as an indicator of growth and stress in a plant as it is positively correlated to leaf chlorophyll content (i.e., photosynthetic activity) and nitrate amount (i.e., availability of main nutrients for plant growth) [47,48,49]. Therefore, the finding of some stress conditions in *Ludwigia* is probably a consequence of its cultivation in an indoor environment, which could not fully replicate the natural and optimal growth conditions for this species; despite this, the alien plant still managed to grow, compete, and negatively affect the growth of the target native plant.

## 3. Materials and Methods

### 3.1. Plant Material

Samples of *L. hexapetala* were collected in May 2022 from a growing population in a channel near Latina in central Italy (41°22′32.0″ N; 13°07′54.0″ E). This population was first reported in 2017 and represented the southernmost population of *L. hexapetala* in Italy [50]. Samples of *U. australis* were taken from a nursery specialised in aquatic plants (Water Nursery, Latina—41°31′35.50″ N; 12°55′10.80″ E). At the sites where the two species were collected, they were not present together. 

### 3.2. Experimental Design

A set of eight glass tanks (25 × 40 × 35 cm, 35 L), similar in size and shape, was set up for the experiment. Each tank was filled with 12 litres of tap water. Four tanks were dedicated to the “plant arena” (U+L) (henceforth arena test) and in each of these three individuals of *U. australis*, along with one of *L. hexapetala*, were placed. Each *U. australis* individual has been carefully selected to have at least one apical bud. In each of the other four tanks, three individuals of *U. australis* were placed in absence of the alien species to be used as control (U). All tanks were placed to receive direct sunlight with a similar intensity throughout the day. The duration of the entire experiment was three weeks, and each test was sampled in the morning at different time points: 0 (T0), 7 (T7), 14 (T14) and 21 (T21) days.

### 3.3. Water Chemical and Physical Analyses

In each tank, measurements of water chemical and physical parameters, such as temperature (T, °C), pH (pH, pH values), conductivity (C, µS/cm), and dissolved oxygen concentration (DO, mg/l), were performed using a multiparametric immersion probe (Hach-Lange HQ40d). In addition, a 50 mL water sample was taken from each tank and stored in sterile plastic tubes at −18 °C to be subsequently analysed for possible presence of allelochemicals. Specifically, quercitrin, a known allelopathic substance produced and released by *L. hexapetala* mainly from the roots [19], was searched via spectrophotometric analysis in the UV-visible range (UV-vis spectrophotometer, Shimadzu 2401 PC). Luminosity above water surface was also registered for each tank in the morning (around 11 a.m.) using a quantum photo/radiometer (Delta Ohm DO 9721).

### 3.4. Plant Parameter Measures

At each time point, some morphological and structural traits of *U. australis* shoots were measured, in particular: total shoot length, number and length of internodes, and number of traps in a 5 cm section immediately below the apical bud. Total shoot length of *Ludwigia* samples was measured using a precision calliper. The number and length of internodes have been considered in literature as reliable traits for assessing the growth and health condition of *Utricularia* species, including *U. australis*, as a higher number of internodes, and longer internodes, indicate an increase in biomass produced, i.e. that vegetative growth has occurred [51,52]. 

The chlorophyll index (SPAD) was only measured for *L. hexapetala* using a chlorophyll meter (SPAD 502 plus, Konica Minolta, Tokyo, Japan), a widely used portable device for fast, accurate and non-destructive chlorophyll measurements. Such measurements could not be carried out on *Utricularia* individuals because of the capillary structure of their leaves, which would not have allowed reliable data detection by this instrument. Fresh weight (FW) of both plant species was measured using a precision scale (MFD, A&D Company, Tokyo, Japan) after letting samples dry on paper towels for 1 min. Starting from FW values, relative growth rate (RGR) for both species was calculated weekly using the following formula [53]:

RGR = (ln FW_2_ − ln FW_1_)/(T_2_ − T_1_)

where FW_1_ is the total fresh weight at time 1 (T_1_) and FW_2_ at the next time 2 (T_2_).

### 3.5. Statistical Analyses

Variations between different tests (U; U+L) and over time (T0, T7, T14, T21) of the various water and plant parameters considered were statistically analysed, fitting a linear mixed model for each parameter, using test and time as categorical explanatory variables, and considering the individual tanks as a random effect. Linear mixed models were fitted using the lmer function from package lme4 [54]. The best model for each case was selected by comparing AIC (Akaike Information Criterion) values. Normality and homoscedasticity assumptions were verified on model residuals using plots and appropriate tests (Shapiro–Wilk for normality and Fligner–Levene for homoscedasticity). Data concerning the number of internodes were log-transformed to meet assumptions of homoscedasticity. Marginal and conditional pseudo-R^2^ for each model were calculated using the R package MuMIn [55]. Marginal R2 represents the proportion of variance explained by the fixed effects; conditional R2 represents the proportion of variance explained by the entire model, including fixed and random effects [55]. The effect of fixed factors in models was tested using Satterthwaite’s method. All statistical analyses were performed with R software vers. 4.2.1 [56]. Plots were made using ggplot2 package [57].

## 4. Conclusions

The results of this laboratory study suggest that the alien *L. hexapetala* appears to impact the native *U. australis* both directly by limiting its growth and affecting the regular development of some of its structural traits (shoot length, internode number and length, trap number), and indirectly, by deteriorating the water quality of the environment in which *U. australis* grows, reducing available dissolved oxygen and altering conductivity and pH levels. Furthermore, it should be noted that *L. hexapetala* exerted a negative influence on this native species even though it had low percentage coverage and it was not in its optimal growing conditions (due to intrinsic limitations of the indoor experiments). Therefore, it is safe to assume that the extent of the impact that *Ludwigia* exerted on this native species in this experiment is an underestimate of what might actually occur in the field in those sites where the alien has greater coverage and is in a condition to express its full invasive potential. Indeed, the impact of an invasive alien species on other plant species is generally associated with its degree of dominance in the invaded area [58].

In order to fully understand the impact that this alien species may have on native aquatic species, investigations carried out directly in the field would be necessary, and to supplement the results obtained from indoor experiments, such as this one (based on a single population of the alien species and of relatively short duration—21 days), with field data where the alien species grows under optimal natural conditions and can best express its invasive potential. 

## Figures and Tables

**Table 1:** Marginal R^2^, conditional R^2^ and *p* values calculated for linear mixed models relative to each parameter. Test *p* value refers to difference between control and plant arena tests; time *p* value refers to differences over time during the experiment; interaction *p* value refers to interaction between test and time. *Ludwigia* parameters do not have different tests to compare between so the only significant difference can be inferred over time (hence the NAs). NS stands for not significant, indicating that the term was removed during model selection when fitting the LMM since it was not significant.

Parameter	Marginal R^2^	Conditional R^2^	Test *p* Value	Time *p* Value	Interaction *p* Value
Dissolved oxygen	0.9012	0.9648	0.0006 *	5.8 × 10^−12^ *	3.3 × 10^−7^ *
Conductivity	0.9718	0.9897	2.9 × 10^−5^ *	2.2 × 10^−16^ *	5.9 × 10^−13^ *
pH	0.9800	0.9804	7.6 × 10^−6^ *	2.2 × 10^−16^ *	3.1 × 10^−5^ *
*Utricularia* total shoot length	0.3213	0.7991	0.3647	0.2749	0.0003 *
*Utricularia* FW	0.7906	0.8750	0.0033 *	8.5 × 10^−5^ *	0.0119 *
*Utricularia* RGR	0.5035	0.5035	0.0011 *	0.3111	N.S.
*Utricularia* internode number	0.2605	0.8623	0.7187	0.0001 *	0.0039 *
*Utricularia* internode length	0.7890	0.7890	1.2 × 10^−5^ *	1.5 × 10^−7^ *	0.2605
*Utricularia* trap number	0.4271	0.4793	0.0283 *	0.0124 *	N.S.
*Ludwigia* shoot length	0.5887	0.7007	NA	0.0033 *	NA
*Ludwigia* FW	0.5842	0.9697	NA	3.7 × 10^−7^ *	NA
*Ludwigia* RGR	0.0555	0.0555	NA	0.7317	NA
*Ludwigia* SPAD	0.5417	0.6707	NA	0.0060 *	NA

* Significant *p* values.

## Data Availability

Not applicable.
